# Adjuvant Chemotherapy Outcomes in Older Adults With Nonmetastatic Triple-Negative Breast Cancer

**DOI:** 10.1001/jamanetworkopen.2026.5061

**Published:** 2026-04-06

**Authors:** Jesus D. Anampa, Jorge Avila, Michael Brodsky, Emery Hakim, Samilia Obeng-Gyasi, Alvaro Alvarez, Carolina Bernabe-Ramirez, Xiaonan Xue

**Affiliations:** 1 Department of Medical Oncology, Montefiore Einstein Comprehensive Cancer Center, Bronx, New York; 2 Cancer Dormancy Institute, Albert Einstein College of Medicine, Bronx, New York; 3 Department of Medicine, Hematology Oncology Fellowship Program, Montefiore Health System, Bronx, New York; 4 Medical School, Albert Einstein College of Medicine, Bronx, New York; 5 Division of Surgical Oncology, Department of Surgery, The Ohio State University, Columbus; 6 Department of Oncology, Brown University Health Cancer Institute, Providence, Rhode Island; 7 Department of Epidemiology & Population Health, Albert Einstein College of Medicine, Bronx, New York

## Abstract

**Importance:**

Triple-negative breast cancer (TNBC) is an aggressive subtype of breast cancer for which current guidelines recommend adjuvant chemotherapy. Data about the benefit of adjuvant chemotherapy in older women with TNBC are scarce.

**Objective:**

To assess the survival benefit of adjuvant chemotherapy in older women with nonmetastatic TNBC.

**Design, Setting, and Participants:**

This retrospective population-based cohort study used the Surveillance, Epidemiology, and End Results database from January 1, 2010, to December 31, 2021. Participants included women 70 years or older with nonmetastatic TNBC who underwent surgical removal of breast tumor and were candidates for adjuvant chemotherapy. The median follow-up was 46 (IQR, 21-83) months. Statistical analysis was conducted on August 4, 2025.

**Exposure:**

Receipt or nonreceipt of adjuvant chemotherapy.

**Main Outcomes and Measures:**

The primary outcome was breast cancer–specific survival between patients who received chemotherapy and those who did not. Logistic regression models assessed variables associated with chemotherapy use. Machine learning with a generalized boosted model was used to estimate propensity scores. Inverse probability of treatment weighting and Cox proportional hazards regression methods were used to compare breast cancer–specific and overall survival.

**Results:**

A total of 5730 women (median age, 76 [IQR, 73-81] years) were included in the analysis; 2509 received chemotherapy and 3221 did not. Patients who received chemotherapy were younger (median age, 74 [IQR, 71-77] vs 79 [IQR, 74-84] years) and had more advanced disease (stage II-III, 1388 [54%.5] vs 1664 [51.7%]) than those who did not. Adjuvant chemotherapy was associated with improved breast cancer–specific survival (hazard ratio [HR], 0.69; 95% CI, 0.58-0.82) and overall survival (HR, 0.55; 95% CI, 0.49-0.62). Similar results were observed across subgroups. Increasing age (odds ratio [OR] for 80-89 years, 0.15 [95% CI, 0.13-0.17]; OR for ≥90 years, 0.02 [95% CI, 0.01-0.04]) was associated with lower odds of receiving adjuvant chemotherapy.

**Conclusions and Relevance:**

In this cohort study of older women with TNBC, adjuvant chemotherapy was associated with improved survival outcomes. Underutilization of adjuvant chemotherapy in older women may contribute to worse outcomes. Geriatric assessment tools may help guide individualized treatment decisions and promote equitable care.

## Key Points

**Question:**

Among older women with nonmetastatic triple-negative breast cancer, is adjuvant chemotherapy associated with better survival outcomes compared with no chemotherapy?

**Findings:**

In this cohort study of 5730 patients 70 years or older with nonmetastatic triple-negative breast cancer, receipt of adjuvant chemotherapy was associated with improved breast cancer–specific survival and overall survival compared with no chemotherapy.

**Meaning:**

These findings suggest that even among older patients—who may be more vulnerable to toxic effects of treatment—chemotherapy can meaningfully extend life and reduce the risk of death from breast cancer.

## Teaser

This cohort study assessess whether adjuvant chemotherapy is associated with breast cancer mortality among women 70 years or older with nonmetastatic triple-negative breast cancer.

## Introduction

Breast cancer mortality has declined by 40% during the last 4 decades, which is partially attributable to improved systemic treatment for early-stage breast cancer.^1,2^ The risk of developing breast cancer increases with advancing age, with women 70 years or older representing only one-third of incident breast cancer cases annually; however, they account for 47% of all breast cancer–specific deaths.^3^ Thus, breast cancer in women 70 years or older is a major public health issue, especially as the US older adult population is expected to grow substantially in the upcoming decades.^4,5^

Approximately 10% of new breast cancers in older women are triple-negative breast cancer (TNBC), an aggressive subtype that lacks estrogen receptor (ER) and progesterone receptor (PR) expression and *ERBB2* (formerly human epidermal growth factor receptor 2 [*HER2*]) expression or amplification.^6,7^ Because antihormone treatment and *ERBB2*-directed therapies are ineffective in early-stage TNBC, multiagent chemotherapy is frequently indicated.^8,9^ Adjuvant chemotherapy in older women poses challenges. Chemotherapy can be associated with serious toxic effects, decline in functional status,^10^ and drug-drug interactions that can result in undertreatment of breast cancer in this population.^11,12^

In addition to toxicity concerns, there are also concerns about the efficacy of adjuvant chemotherapy in this population.^13,14,15^ Most prospective studies assessing the benefit of chemotherapy in early-stage breast cancer did not include many older women; therefore, data about the benefit of adjuvant chemotherapy in this population are scarce.^16,17^ We aimed to assess the benefit of adjuvant chemotherapy on breast cancer mortality among women 70 years or older with nonmetastatic TNBC.

## Methods

### Study Design and Data Collection

We conducted a retrospective cohort study using the Surveillance, Epidemiology, and End Results (SEER) database. Inclusion criteria consisted of (1) female sex, (2) age 70 years or older, (3) breast cancer diagnosed between January 1, 2010, and December 31, 2021, (4) negative for ER, PR, and *ERBB2*; (5) ductal histology (*International Classification of Disease for Oncology, Third Edition*, code 8500/3); (6) no prior neoadjuvant therapy; (7) surgical removal of breast tumor; and (8) candidacy for adjuvant chemotherapy (node negative with tumor size >5 mm or node positive with any tumor size). Exclusion criteria consisted of ductal carcinoma in situ, metastatic disease, prior malignant disease, and unknown tumor size or nodal status. The deidentified data were determined to be exempt from informed consent by the Institutional Review Board of the Albert Einstein College of Medicine, which approved the study. The study adhered to the Strengthening the Reporting of Observational Studies in Epidemiology (STROBE) reporting guideline.

### Variables

We collected data on demographic variables such as diagnosis year, age, and race and ethnicity. Race data were obtained from the SEER database and then categorized in combination with ethnicity data to comprise 4 groups: Hispanic, non-Hispanic Black, non-Hispanic White, and non-Hispanic other (including non-Hispanic American Indian or Alaska Native and non-Hispanic Asian or Pacific Islander). Race and ethnicity data were collected because prior studies have reported racial disparities in breast cancer survival outcomes; therefore, this variable needed to be included in the survival analysis of older women with breast cancer. Socioeconomic variables included rurality, marital status, and median household income. Breast cancer clinicopathologic variables included tumor size, nodal status, pathologic stage, and tumor grade. Treatment variables included radiotherapy receipt, type of surgery (breast-conserving surgery vs mastectomy), and chemotherapy use.

### Statistical Analysis

Based on chemotherapy receipt, we divided eligible patients into 2 groups: those who received chemotherapy and those who did not. Descriptive statistics were used to summarize the baseline characteristics. The Wilcoxon rank sum test was used to compare continuous variables, and the χ^2^ test was used to compare categorical variables.

Logistic regression models were used to assess factors associated with chemotherapy administration; adjusted odds ratios (ORs) with 95% CIs were obtained from these models. Breast cancer–specific survival (BCSS), our primary end point, was defined as the time in months from diagnosis to death from breast cancer or the last follow-up for censored patients. To examine the effect of chemotherapy on the breast cancer–specific death rate, patients who died from causes other than breast cancer were censored at their date of death in the BCSS analysis.

We used inverse probability of treatment weighting (IPTW)^18,19^ to account for differences in patient characteristics between the 2 groups. We adopted a machine learning method, the generalized boosted model, to estimate the probability of treatment (ie, the propensity score). Variables for the propensity score included age, race and ethnicity, cancer stage, tumor grade, radiotherapy, type of surgery, marital status, rurality, income, and diagnosis year. Several studies^20,21,22,23^ have found that among a variety of propensity score estimation methods, the generalized boosted model provides estimated weights that yield the best balance between treatment groups and estimated the treatment effect with the smallest mean squared error.^20,24^ Extreme weight values can inflate the variance and 95% CIs of the effect; therefore, weights were truncated at the 1st and 99th percentiles.^19^ Balance diagnostics showed that the weighting process successfully removed differences in baseline covariates (eFigure 1 and eTable 1 in Supplement 1). BCSS was assessed using weighted Cox proportional hazards regression models with a robust sandwich variance estimator to account for the lack of independent observations induced by the weighting process.^25^ Hazard ratios (HRs) and 95% CIs were estimated from these models. Then, stratified analyses were performed to examine the difference in BCSS for cohorts with and without chemotherapy by age, race and ethnicity, tumor grade, cancer stage, radiotherapy, and type of surgery.^26^ The interaction between chemotherapy and other prognostic factors was assessed by including a cross-product term in the multivariable-adjusted models. The Wald test was used to assess the interaction effects. We also examined the effect of chemotherapy on the cumulative incidence rate of death from breast cancer over time, accounting for competing risks due to other deaths.^27,28^

Overall survival (OS) was defined as the time in months from diagnosis to death from any cause or last follow-up for censored patients. We estimated OS for the cohorts receiving and not receiving chemotherapy using Kaplan-Meier methods. We then created IPTW-adjusted Cox proportional hazards regression models, as described for BCSS, to compare OS for the groups with and without chemotherapy in the overall cohort and subgroup analysis.

Case-complete statistical analysis was conducted on August 4, 2025. Two-sided *P* values and 95% CIs are reported. α = .05 was used for all hypothesis testing to indicate statistical significance. Statistical analyses were performed in R, version 1.4.1106 (R Project for Statistical Computing).

## Results

Among the 5730 female patients included in our study (median age, 76 [IQR, 73-81] years), 2509 received chemotherapy and 3221 did not (eFigure 2 in Supplement 1). Patients treated with adjuvant chemotherapy were younger (median age, 74 [IQR, 71-77] vs 79 [IQR, 74-84] years) and had more advanced disease (stage II-III, 1388 [54.5%] vs 1664 [51.7%]) and higher tumor grade (poorly differentiated or undifferentiated tumors, 2052 [81.8%] vs 2434 [75.6%]) than those who did not receive adjuvant chemotherapy. Breast-conserving surgery (1683 [67.1%] vs 1786 [55.4%]) and adjuvant radiotherapy (1515 [60.4%] vs 1394 [43.3%]) were used more frequently among patients who received adjuvant chemotherapy. Baseline characteristics are illustrated in Table 1.

**Table 1.** Baseline Characteristics of Older Patients With Stages I to III Triple-Negative Breast Cancer

Characteristic	Patient group			*P* value
	Overall (N = 5730)	Chemotherapy (n = 2509)	No chemotherapy (n = 3221)	
Age, median (IQR), y	76 (73-81)	74 (71-77)	79 (74-84)	<.001
Age group, y				
70-79	3883 (67.8)	2209 (88.0)	1674 (52.0)	<.001
80-89	1666 (29.1)	295 (11.8)	1371 (42.6)	
≥90	181 (3.2)	5 (0.2)	176 (5.5)	
Year of diagnosis				
2010-2013	2007 (35.0)	801 (31.9)	1206 (37.4)	<.001
2014-2017	1915 (33.4)	836 (33.3)	1079 (33.5)	
2018-2021	1808 (31.6)	872 (34.8)	936 (29.1)	
Race and ethnicity				
Hispanic	481 (8.4)	200 (8.0)	281 (8.7)	.41
Non-Hispanic Black	875 (15.3)	402 (16.0)	473 (14.7)	
Non-Hispanic White	3930 (68.6)	1718 (68.5)	2212 (68.7)	
Non-Hispanic other^a^	444 (7.7)	189 (7.5)	255 (7.9)	
Tumor stage				
T1	3066 (53.5)	1362 (54.3)	1704 (52.9)	<.001
T2	2249 (39.2)	1028 (41.0)	1221 (37.9)	
T3	268 (4.7)	87 (3.5)	181 (5.6)	
T4	147 (2.6)	32 (1.3)	115 (3.6)	
Nodal stage				
N0	4215 (73.6)	1781 (71.0)	2434 (75.6)	<.001
N1	1059 (18.5)	515 (20.5)	544 (16.9)	
N2	278 (4.9)	136 (5.4)	142 (4.4)	
N3	178 (3.1)	77 (3.1)	101 (3.1)	
Cancer stage				
I	2698 (47.1)	1141 (45.5)	1557 (48.3)	<.001
II	2227 (38.9)	1035 (41.3)	1192 (37.0)	
III	805 (14.0)	333 (13.3)	472 (14.7)	
Tumor grade				
Well differentiated	109 (1.9)	30 (1.2)	79 (2.5)	<.001
Moderately differentiated	1135 (19.8)	427 (17.0)	708 (22.0)	
Poorly differentiated or undifferentiated	4486 (78.3)	2052 (81.8)	2434 (75.6)	
Marital status				
Single	521 (9.1)	242 (9.6)	279 (8.7)	<.001
Prior married	2564 (44.7)	937 (37.3)	1627 (50.5)	
Married	2392 (41.7)	1250 (49.8)	1142 (35.5)	
Unknown	253 (4.4)	80 (3.2)	173 (5.4)	
Rurality				
Metropolitan	4994 (87.2)	2181 (86.9)	2813 (87.3)	.68
Nonmetropolitan	736 (12.8)	328 (13.1)	408 (12.7)	
Income				
<$45 000	219 (3.8)	107 (4.3)	112 (3.5)	.02
$45 000-$54 999	521 (9.1)	232 (9.2)	289 (9.0)	
$55 000-$64 999	795 (13.9)	346 (13.8)	449 (13.9)	
$65 000-$74 999	1200 (20.9)	477 (19.0)	723 (22.4)	
≥$75 000	2995 (52.3)	1347 (53.7)	1648 (51.2)	
Radiotherapy				
No	2821 (49.2)	994 (39.6)	1827 (56.7)	<.001
Yes	2909 (50.8)	1515 (60.4)	1394 (43.3)	
Surgery type				
Breast-conserving surgery	3469 (60.5)	1683 (67.1)	1786 (55.4)	.01
Mastectomy	2261 (39.5)	826 (32.9)	1435 (44.6)	

^a^ Includes non-Hispanic American Indian or Alaska Native and non-Hispanic Asian or Pacific Islander.

**Figure 1.**
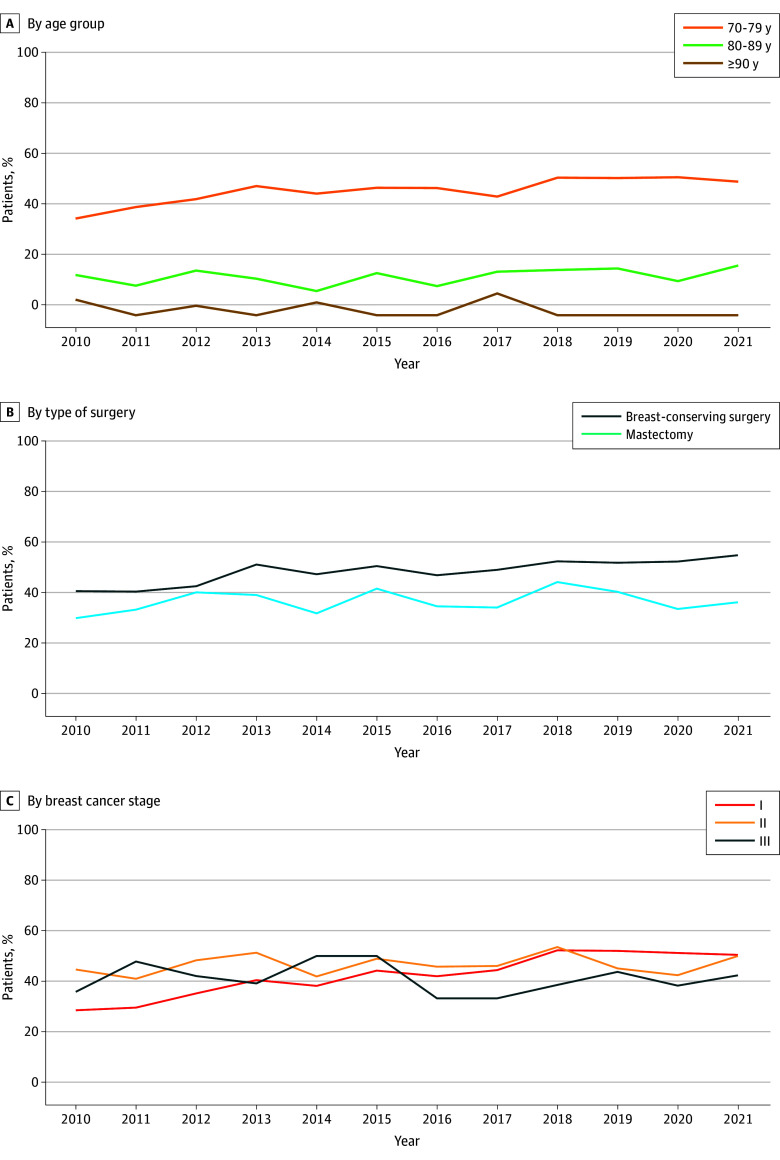
Line Graphs Showing Adjuvant Chemotherapy Use Over Time by Age, Type of Surgery, and Cancer Stage

The use of adjuvant chemotherapy increased during our study period (179 of 500 [35.8%] in 2010 vs 218 of 448 [48.7%] in 2021; *P* < .001). This increase was evidenced among patients aged 70 to 79 years (151 of 340 [44.4%] in 2010 vs 189 of 308 [61.4%] in 2021; *P* < .001), but not among those aged 80 to 89 years or 90 years or older (Figure 1 and eTable 2 in Supplement 1). The use of adjuvant chemotherapy increased among those who underwent breast-conserving surgery (111 of 273 [40.7%] in 2010 vs 164 of 299 [54.8%] in 2021; *P* < .001), whereas there was no change among those who underwent mastectomy (Figure 1). During the study period, the use of adjuvant chemotherapy increased in patients with stage I (67 of 234 [28.6%] in 2010 vs 112 of 222 [50.5%] in 2021; *P* < .001), whereas there was no change for those with stage II (84 of 188 [44.7%] in 2010 vs 67 of 134 [50.0%] in 2021) and stage III (28 of 78 [35.9%] in 2010 vs 39 of 92 [42.4%] in 2021) breast cancer (Figure 1).

### Factors Associated With Chemotherapy Administration

Increasing age was associated with lower odds of receiving adjuvant chemotherapy (OR for 80-89 years, 0.15 [95% CI, 0.13-0.17]; OR for ≥90 years, 0.02 [95% CI, 0.01-0.04]). Higher tumor grade (OR for poorly differentiated or undifferentiated, 2.35; 95% CI, 1.50-3.74) and advanced cancer stage (OR for stage III, 1.96; 95% CI, 1.60-2.40) were associated with increased odds of adjuvant chemotherapy. Those who underwent mastectomy had lower odds of adjuvant chemotherapy (OR, 0.83; 95% CI, 0.71-0.97), whereas those who received adjuvant radiotherapy had higher odds of adjuvant chemotherapy (OR, 1.77; 95% CI, 1.54-2.04). The odds of chemotherapy did not vary among racial groups (Table 2).

**Table 2.** Factors Associated With Administration of Adjuvant Chemotherapy in Older Patients With Stages I to III Triple-Negative Breast Cancer

Characteristic	OR (95% CI)	*P* value
Age group, y		
70-79	1 [Reference]	NA
80-89	0.15 (0.13-0.17)	<.001
≥90	0.02 (0.01-0.04)	<.001
Race and ethnicity		
Hispanic	0.92 (0.74-1.15)	.47
Non-Hispanic Black	0.99 (0.84-1.17)	.94
Non-Hispanic White	1 [Reference]	NA
Non-Hispanic other^a^	1.05 (0.84-1.32)	.65
Year of diagnosis		
2010-2015	1 [Reference]	NA
2016-2017	1.06 (0.90-1.25)	.48
2018-2021	1.31 (1.14-1.50)	<.001
Marital status		
Never married	1 [Reference]	NA
Prior married	0.81 (0.66-1.00)	.05
Married	1.14 (0.92-1.41)	.22
Unknown	0.54 (0.38-0.77)	<.001
Rurality		
Metropolitan	1 [Reference]	NA
Nonmetropolitan	1.03 (0.84-1.24)	.80
Income		
<$70 000	1 [Reference]	NA
≥$70 000	1.03 (0.90-1.18)	.68
Tumor grade		
Well differentiated	1 [Reference]	NA
Moderately differentiated	1.67 (1.06-2.70)	.03
Poorly differentiated or undifferentiated	2.35 (1.50-3.74)	<.001
Cancer stage		
I	1 [Reference]	NA
II	1.81 (1.59-2.07)	<.001
III	1.96 (1.60-2.40)	<.001
Surgery		
Breast-conserving surgery	1 [Reference]	NA
Mastectomy	0.83 (0.71-0.97)	.02
Radiotherapy		
No	1 [Reference]	NA
Yes	1.77 (1.54-2.04)	<.001

Abbreviations: NA, not applicable; OR, odds ratio.

^a^ Includes non-Hispanic American Indian or Alaska Native and non-Hispanic Asian or Pacific Islander.

### Breast Cancer Mortality

In a multivariate Cox proportional hazards regression model with IPTW, chemotherapy administration was associated with improved BCSS (HR, 0.69; 95% CI, 0.58-0.82; *P* < .001) (eTable 3 in Supplement 1). Adjuvant radiotherapy was associated with better BCSS (HR, 0.70; 95% CI, 0.58-0.85), whereas increasing age (HR for ≥90 years, 1.99; 95% CI, 1.40-2.84), high tumor grade (HR for undifferentiated or poorly differentiated, 1.41; 95% CI, 1.13-1.76), and advanced cancer stage (HR for stage III, 8.60; 95% CI, 6.74-10.96) were associated with worse BCSS.

We further analyzed the chemotherapy-associated BCSS according to demographic, clinicopathologic, and treatment covariables. The association of adjuvant chemotherapy with improved BCSS varied with age (*P* = .01 for interaction). Among patients aged 70 to 79 years (HR, 0.65; 95% CI, 0.54-0.80) and 80 to 89 years (HR, 0.71; 95% CI, 0.52-0.98), chemotherapy was associated with improved BCSS; however, there was no difference in BCSS among patients 90 years or older who received chemotherapy vs those who did not. The association of adjuvant chemotherapy with improved BCSS was similar across all subgroups for race and ethnicity compared with non-Hispanic White patients (HR for non-Hispanic Black, 1.02 [95% CI, 0.64-1.64]; HR for Hispanic, 1.50 [95% CI, 0.80-2.80]; HR for non-Hispanic other race, 1.28 [95% CI, 0.61-2.67]; *P* = .59 for interaction), tumor grade (HR for poorly differentiated or undifferentiated vs well or moderately differentiated, 0.87; [95% CI, 0.55-1.40]; *P* = .58 for interaction), cancer stage compared with stage I (HR for stage II, 0.96 [95% CI, 0.63-1.46]; HR for stage III, 0.75 [95% CI, 0.46-1.20; *P* = .39 for interaction), radiotherapy (HR for radiotherapy vs no radiotherapy, 0.75 [95% CI, 0.54-1.06]; *P* = .10 for interaction), and surgery (HR for mastectomy vs breast conserving surgery, 0.99 [95% CI, 1.71-1.38]; *P* = .94 for interaction) (Table 3).

**Table 3.** Association of Chemotherapy With Improved Breast Cancer–Specific Survival for Older Patients With Stage I to III Triple-Negative Breast Cancer After Inverse Probability of Treatment Weighting Analysis^a^

Subgroup	No. (%) of patients	No. (%) of events	HR (95% CI)	5-y Breast cancer death risk by chemotherapy receipt, %		*P* value for interaction
				No	Yes	
Overall	5730 (100)	838 (100)	0.69 (0.58-0.82)	18	13	NA
Age, y						
70-79	3883 (67.8)	465 (55.5)	0.65 (0.54-0.80)	14	12	.01
80-89	1666 (29.1)	319 (38.1)	0.71 (0.52-0.98)	22	21	
≥90	181 (3.2)	54 (6.4)	2.49 (0.62-10.01)	31	47	
Race and ethnicity						
Hispanic	481 (8.4)	68 (8.1)	1.001 (0.52-1.92)	15	17	.59
Non-Hispanic Black	875 (15.3)	135 (16.1)	0.64 (0.42-0.98)	20	15	
Non-Hispanic White	3930 (68.6)	587 (70.0)	0.66 (0.53-0.81)	19	13	
Non-Hispanic other^b^	444 (7.7)	48 (5.7)	0.73 (0.34-1.56)	13	10	
Tumor grade						
Well or moderately differentiated	1244 (21.7)	122 (14.6)	0.78 (0.47-1.29)	10	9	.58
Poorly differentiated or undifferentiated	4486 (78.3)	716 (85.4)	0.69 (0.57-0.83)	20	14	
Cancer stage						
I	2698 (47.1)	169 (20.2)	0.78 (0.54-1.12)	7	6	.39
II	2227 (38.9)	380 (45.3)	0.76 (0.59-0.97)	21	15	
III	805 (14.0)	289 (34.5)	0.61 (0.45-0.83)	50	35	
Radiotherapy						
No	2821 (49.2)	526 (62.8)	0.77 (0.63-0.96)	21	17	.10
Yes	2909 (50.8)	312 (37.2)	0.57 (0.43-0.73)	14	11	
Surgery						
Mastectomy	2261 (39.5)	493 (58.8)	0.67 (0.54-0.86)	25	20	.94
Breast-conserving surgery	3469 (60.5)	345 (41.2)	0.68 (0.53-0.88)	12	10	

Abbreviations: HR, hazard ratio; NA, not applicable.

^a^ Models were adjusted for race and ethnicity, age, cancer stage, tumor grade, radiotherapy, type of surgery, household income, marital status, rurality, and year of diagnosis.

^b^ Includes non-Hispanic American Indian or Alaska Native and non-Hispanic Asian or Pacific Islander.

**Figure 2.**
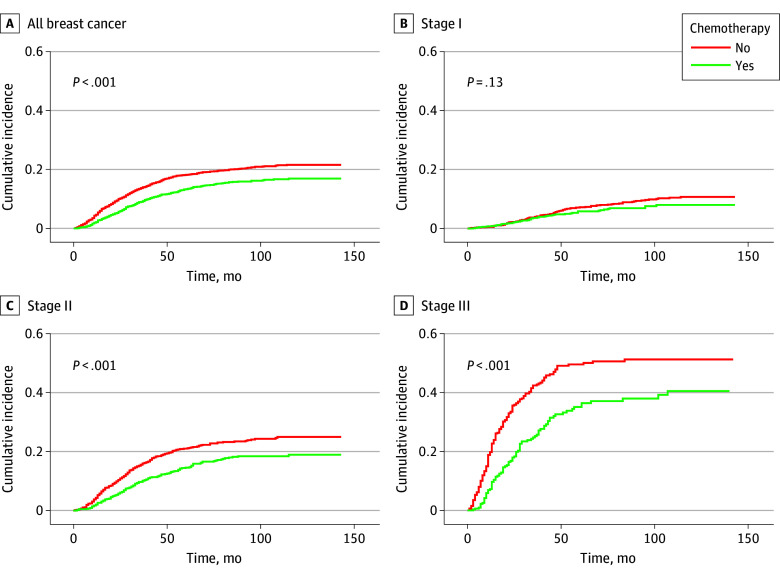
Line Graphs Showing Cumulative Incidence of Breast Cancer Death by Chemotherapy Use

During the study period, 838 women (14.6%) died due to breast cancer and 1142 (19.9%) died due to other causes. The 5-year cumulative risk of breast cancer death was lower for those who received chemotherapy compared with those who did not (13.3% vs 18.1%). When stratified by cancer stage, chemotherapy was associated with a decrease in the risk of breast cancer death in stages II and III (21.0% vs 14.6% and 49.6% vs 35.1%, respectively) but not stage I (7.2% vs 5.8%) (Figure 2). The cumulative risk of non–breast cancer death was lower in patients who received adjuvant chemotherapy (5-year risk of non–breast cancer death, 8.1% vs 23.0%). Similar results were evidenced in all cancer stage subgroups (eFigure 3 in Supplement 1).

### Overall Survival

During a median follow-up of 46 (IQR, 21-83) months, 1980 patients (34.6%) with stage I to III TNBC died. The 5-year OS rate was higher for those who received chemotherapy vs those who did not (76.4% vs 60.9%) (eFigure 4 in Supplement 1). The 5-year OS rates for receipt of chemotherapy vs no chemotherapy were 87.9% vs 77.3% in patients with stage I cancer, 73.2% vs 54.0% in patients with stage II cancer, and 45.3% vs 21.9% in patients with stage III cancer.

In a multivariate Cox proportional hazards regression model with IPTW, chemotherapy administration was associated with improved OS (HR, 0.55; 95% CI, 0.49-0.62; *P* < .001) (eTable 4 in Supplement 1). Adjuvant radiotherapy was also associated with better OS (HR, 0.76; 95% CI, 0.66-0.87). Increasing age (HR for ≥90 years, 2.38; 95% CI, 1.92-2.94), high tumor grade (HR for undifferentiated or poorly differentiated, 1.37; 95% CI, 1.19-1.57), and advanced cancer stage (HR for stage III, 4.43; 95% CI, 3.73-5.25) were associated with worse OS.

Next, we analyzed the chemotherapy-associated OS according to demographic, clinicopathologic, and treatment covariables. The association of adjuvant chemotherapy with improved OS was similar across all subgroups for age compared with 70 to 79 years (HR for 80-89 years, 0.99 [95% CI, 0.77-1.27]; HR for ≥90 years, 1.34 [95% CI, 0.71-2.51]; *P* = .65 for interaction), race and ethnicity compared with non-Hispanic White patients (HR for non-Hispanic Black, 1.12 [95% CI, 0.83-1.51]; HR for Hispanic, 1.33 [95% CI, 0.89-1.99]; HR for non-Hispanic other race, 1.05 [95% CI, 0.64-1.73]; *P* = .53 for interaction), tumor grade (HR for poorly differentiated or undifferentiated vs well or moderately differentiated, 0.92 [95% CI, 0.68-1.26]; *P* = .62 for interaction), cancer stage compared with stage I (HR for stage II, 0.94 [95% CI, 0.73-1.21]; HR for stage III, 0.84 [95% CI, 0.60-1.18]; *P* = .60 for interaction), radiotherapy (HR for radiotherapy vs no radiotherapy, 0.91 [95% CI, 0.73-1.15]; *P* = .45 for interaction), and surgery (HR for mastectomy vs breast conserving surgery, 1.04 [95% CI, 0.83-1.31]; *P* = .72 for interaction) (eTable 5 in Supplement 1).

## Discussion

In this large population study, we found that when adjusted for demographic characteristics and clinicopathologic, treatment, and socioeconomic covariables, adjuvant chemotherapy was associated with improved BCSS (HR, 0.69; 95% CI, 0.58-0.82; *P* < .001) and OS (HR, 0.55; 95% CI, 0.49-0.62; *P* < .001) in older women with nonmetastatic TNBC. Furthermore, the use of adjuvant chemotherapy has increased from 35.8% in 2010 to 48.7% in 2021 in older women with TNBC. Despite worse BCSS and OS evidenced with increasing age, increasing age was associated with lower odds of adjuvant chemotherapy.

Recent studies have demonstrated a decrease in the use of adjuvant chemotherapy for early-stage breast cancer. Bhimani et al^29^ analyzed the Kaiser Permanente database and reported a decline in receipt of chemotherapy over time from 2006 to 2019 (40.2% vs 35.6%; *P* < .001). However, a study of node-negative, small-size TNBC^30^ reported an increase in chemotherapy use from 2010 to 2019 in patients with T1b (52.5% vs 60.5%; *P* = .001) and T1c (63% vs 71%; *P* < .001) tumors, but no difference for T1a tumors (23.2% vs 19.9%; *P* = .63). Our study showed an increase in the use of adjuvant chemotherapy for older patients with early-stage TNBC; however, this increase was driven by patients with stage I TNBC, with no change for those with stage II or III TNBC. Despite guidelines recommending its use for most patients, chemotherapy was used only in 44.7% to 50.0% of patients with stage II and 35.9% to 42.4% of patients with stage III TNBC during the study period. These low rates of adjuvant chemotherapy may play an important role in the poor outcomes for older patients with stage II or III TNBC.

In our study, despite similar chemotherapy benefit in BCSS, patients who underwent mastectomy had lower odds of chemotherapy use compared with those who underwent breast-conserving surgery. Traditionally, escalation surgery with mastectomy can spare adjuvant radiotherapy in patients with early-stage breast cancer^31^; however, it should not affect the use of other adjuvant therapies such as chemotherapy or endocrine therapy. Additionally, the benefit of adjuvant chemotherapy was evidenced regardless of adjuvant radiotherapy, highlighting that the use of adjuvant radiotherapy should not spare adjuvant chemotherapy for those who need it. Furthermore, there was no benefit of adjuvant chemotherapy in patients 90 years or older, which could be attributable to limited statistical power in this subgroup, competing mortality, or both. The unadjusted Kaplan-Meier analysis showed that chemotherapy was associated with improved OS in stage I TNBC, but not with BCSS in this subgroup. However, the adjusted Cox proportional hazards regression model with propensity score weighting indicated a associated with chemotherapy in BCSS and OS across all cancer stages. Despite the similar level of relative benefit of adjuvant chemotherapy on breast cancer mortality across all breast cancer stages, the absolute benefit varies because the risk of breast cancer death differs. The improvement in 5-year cumulative risk of breast cancer death was larger in patients with stage III cancer, with a 15–percentage point improvement (50% vs 35%), whereas the diffrence was 6 percentage points in stage II (21% vs 15%), and 1 percentage point in stage I (7% vs 6%) (Table 3). Therefore, the decision about adjuvant chemotherapy should be based on a comprehensive discussion about risks and benefits, but also should involve patient comfort level with such absolute benefits or risks, since patients vary in the value they place on potential benefits of a toxic treatment such as chemotherapy.^32,33^ In our study, adjuvant chemotherapy was also associated with improved cumulative risk of non–breast cancer death (5-year risk: 8% vs 23%), which suggests the presence of healthy-user bias, where patients who received adjuvant chemotherapy had fewer comorbidities than those who did not.

The reasons leading to decreased use of chemotherapy with increasing age remain uncertain. Aging can result in alterations in metabolism and distribution of chemotherapy due to changes in liver and kidney function and hemoglobin concentration, which can increase the risks of toxic effects resulting in dose reductions in older patients.^34,35,36,37^ Tools to assess chemotherapy risks in older women have been developed. The Geriatric Assessment (GA) evaluates multiple domains, including functional status, comorbidity, cognition, depression, social activity and support, and nutritional status,^38^ that have predictive value for chemotherapy toxicity that is not necessarily captured by history and physical examination results or simplified performance status scales such as Eastern Cooperative Oncology Group and Karnofsky. Based on the results of the HOPE (Hurria Older Patients With Breast Cancer) trial, a predictive model (Cancer and Aging Research Group–Breast Cancer [CARG-BC] score) was developed for chemotherapy toxicity in the older women, which incorporated use of anthracyclines, stage II or III cancer, planned treatment duration greater than 3 months, abnormal liver function, low hemoglobin levels, falls, limited walking ability, and lack of social support. The CARG-BC model was significantly better at predicting grades 3 to 5 toxic effects from chemotherapy than the Karnofsky scale (areas under the receiver operating characteristic curve, 0.72 [*P* < .001] vs 0.53 [*P* = .19]).^39,40^ The fact that the CARG-BC model, which incorporates GA domains, was better at predicting toxic effects highlights the importance of using a more comprehensive assessment tool that accounts for the diversity of risk factors in older women. Similarly, Cohen et al^41^ incorporated GA domains into a deficit-accumulation frailty index that demonstrated a positive association with increased scores and likelihood to discontinue chemotherapy (relative risk [RR], 2.06; 95% CI, 1.26-3.38; *P* < .01) and to be hospitalized (RR, 1.98; 95% CI, 1.26-3.11; *P* < .01).

The GA-guided management cannot only aid oncologists in identifying poor candidates for adjuvant chemotherapy, it can also improve communication with patients and increase patient satisfaction, thereby facilitating shared decision-making.^42,43,44^ In the context of TNBC, given the mounting evidence of the OS and BCSS benefit of adjuvant chemotherapy in older women, including our study, more comprehensive tools such as the GA should be implemented to improve access to treatment when appropriate. Novel therapeutic options such as immune checkpoint inhibitors and antibody-drug conjugates have emerged as treatment options for TNBC. Due to the unique toxicity profiles of these drugs, the GA is an important tool to guide treatment decisions when using these drugs in older women.

Due to the KEYNOTE-522 trial reporting improved pathologic and survival outcomes with neoadjuvant pembrolizumab and chemotherapy, most patients receive neoadjuvant rather than adjuvant treatments for nonmetastatic TNBC.^45,46,47^ Most patients in our study were treated prior to the adoption of the KEYNOTE-522 regimen; therefore, ongoing evaluation of data in older populations with regard to chemoimmunotherapy is needed.

### Limitations

Our study has certain limitations. First, despite its larger size, it is a retrospective study subjected to the biases of such observational studies. Second, the SEER database does not provide information about chemotherapy regimen, duration of chemotherapy, or dose modifications, all of which may affect chemotherapy efficacy. Third, we were unable to adjust our analysis for patient comorbidities or polypharmacy, since this information is not available in the SEER database. Fourth, some of the subgroup analyses included few survival events and therefore need to be interpreted with caution. Fifth, we included area-level socioeconomic factors, but we were unable to include individual socioeconomic variables in our analyses.

## Conclusions

In this cohort study, older patients with nonmetastatic TNBC benefited from adjuvant chemotherapy. Less than 50% of older patients with stage II or III TNBC received adjuvant chemotherapy, which could have contributed to the poor survival outcomes in these patients. Geriatric assessment tools can guide the use of adjuvant chemotherapy in older patients.

## Notes

Supplement 1. **eTable 1.** Balance Diagnostics Before and After Inverse Probability of Treatment Weighting (IPTW) **eTable 2.** Use of Adjuvant Chemotherapy in Older Patients With TNBC From 2010 to 2021 **eTable 3.** Univariate and Multivariable Analysis for Breast Cancer-Specific Survival in Older Patients With Stages I-III TNBC After Inverse Probability of Treatment Weighting (IPTW) Analysis **eTable 4.** Univariate and Multivariable Analysis for Overall Survival in Older Patients With Stages I-III TNBC After Inverse Probability of Treatment Weighting (IPTW) Analysis **eTable 5.** Benefit of Chemotherapy in Overall Survival for Older Patients With Stages I-III TNBC After Inverse Probability of Treatment Weighting (IPTW) Analysis **eFigure 1.** Balance Diagnostic Before and After IPTW **eFigure 2.** Strobe Diagram **eFigure 3.** Cumulative Incidence of Non–Breast Cancer Death by Chemotherapy Use in Older Patients With Stages I-III TNBC **eFigure 4.** Overall Survival in Older Patients With Stages I-III TNBC

Supplement 2.
Data Sharing Statement

## References

[1] DeSantis CE, Ma J, Gaudet MM, . Breast cancer statistics, 2019. CA Cancer J Clin. 2019;69(6):438-451. doi:10.3322/caac.21583 31577379

[2] Sung H, Ferlay J, Siegel RL, . Global Cancer Statistics 2020: GLOBOCAN estimates of incidence and mortality worldwide for 36 cancers in 185 countries. CA Cancer J Clin. 2021;71(3):209-249. doi:10.3322/caac.21660 33538338

[3] Freedman RA, Keating NL, Lin NU, . Breast cancer-specific survival by age: worse outcomes for the oldest patients. Cancer. 2018;124(10):2184-2191. doi:10.1002/cncr.31308 29499074 PMC5935594

[4] Vespa J, Armstrong D, Medina L. Demographic turning points for the United States: population projections for 2020 to 2060. US Census Bureau. Report 25-1144. February 2020. Accessed June 4, 2025. https://www.census.gov/content/dam/Census/library/publications/2020/demo/p25-1144.pdf

[5] Shreya D, Fish PN, Du D. Navigating the future of elderly healthcare: a comprehensive analysis of aging populations and mortality trends using National Inpatient Sample (NIS) data (2010-2024). Cureus. 2025;17(3):e80442. doi:10.7759/cureus.80442 40225437 PMC11986089

[6] Dent R, Trudeau M, Pritchard KI, . Triple-negative breast cancer: clinical features and patterns of recurrence. Clin Cancer Res. 2007;13(15, pt 1):4429-4434. doi:10.1158/1078-0432.CCR-06-3045 17671126

[7] Howlader N, Altekruse SF, Li CI, . US incidence of breast cancer subtypes defined by joint hormone receptor and HER2 status. J Natl Cancer Inst. 2014;106(5):dju055. doi:10.1093/jnci/dju055 24777111 PMC4580552

[8] Anampa J, Makower D, Sparano JA. Progress in adjuvant chemotherapy for breast cancer: an overview. BMC Med. 2015;13:195. doi:10.1186/s12916-015-0439-8 26278220 PMC4538915

[9] Lu JY, Alvarez Soto A, Anampa JD. The landscape of systemic therapy for early stage triple-negative breast cancer. Expert Opin Pharmacother. 2022;23(11):1291-1303. doi:10.1080/14656566.2022.2095902 35818711

[10] Ji J, Sun CL, Cohen HJ, Muss HB, Bae M, Sedrak MS. Toxicity risk score and clinical decline after adjuvant chemotherapy in older breast cancer survivors. J Natl Cancer Inst. 2023;115(5):578-585. doi:10.1093/jnci/djad029 36762832 PMC10165485

[11] Zhu W, Perez EA, Hong R, Li Q, Xu B. Age-related disparity in immediate prognosis of patients with triple-negative breast cancer: a population-based study from SEER cancer registries. PLoS One. 2015;10(5):e0128345. doi:10.1371/journal.pone.0128345 26020519 PMC4447406

[12] Anampa J, Sparano JA. Tailoring adjuvant therapy for breast cancer in the elderly: room for improvement. Breast J. 2017;23(3):253-255. doi:10.1111/tbj.12730 27900798

[13] Muss HB, Berry DA, Cirrincione C, ; Cancer and Leukemia Group B Experience. Toxicity of older and younger patients treated with adjuvant chemotherapy for node-positive breast cancer: the Cancer and Leukemia Group B Experience. J Clin Oncol. 2007;25(24):3699-3704. doi:10.1200/JCO.2007.10.9710 17704418

[14] Bravo-Solarte DC, Zhang F, Anampa JD. Assessment of use and impact of chemotherapy in lymph node-negative, T1a triple-negative breast cancer. Clin Breast Cancer. 2023;23(7):763-773.e6. doi:10.1016/j.clbc.2023.08.002 37648557

[15] Carbajal-Ochoa W, Bravo-Solarte DC, Bernal AM, Anampa JD. Benefit of adjuvant chemotherapy in lymph node-negative, T1b and T1c triple-negative breast cancer. Breast Cancer Res Treat. 2024;203(2):257-269. doi:10.1007/s10549-023-07132-6 37833449

[16] Eifel P, Axelson JA, Costa J, . National Institutes of Health consensus development conference statement: adjuvant therapy for breast cancer, November 1-3, 2000. J Natl Cancer Inst. 2001;93(13):979-989. doi:10.1093/jnci/93.13.979 11438563

[17] Early Breast Cancer Trialists’ Collaborative Group (EBCTCG). Effects of chemotherapy and hormonal therapy for early breast cancer on recurrence and 15-year survival: an overview of the randomised trials. Lancet. 2005;365(9472):1687-1717. doi:10.1016/S0140-6736(05)66544-0 15894097

[18] Austin PC. The use of propensity score methods with survival or time-to-event outcomes: reporting measures of effect similar to those used in randomized experiments. Stat Med. 2014;33(7):1242-1258. doi:10.1002/sim.5984 24122911 PMC4285179

[19] Austin PC, Stuart EA. Moving towards best practice when using inverse probability of treatment weighting (IPTW) using the propensity score to estimate causal treatment effects in observational studies. Stat Med. 2015;34(28):3661-3679. doi:10.1002/sim.6607 26238958 PMC4626409

[20] McCaffrey DF, Ridgeway G, Morral AR. Propensity score estimation with boosted regression for evaluating causal effects in observational studies. Psychol Methods. 2004;9(4):403-425. doi:10.1037/1082-989X.9.4.403 15598095

[21] Ridgeway G. Generalized Boosted Models: A guide to the gbm package. January 22, 2026. Accessed July 31, 2025. https://cran.r-project.org/web/packages/gbm/vignettes/gbm.pdf

[22] Friedman JH. Greedy function approximation: a gradient boosting machine. Ann Statist. 2001;29(5):1189-1232. doi:10.1214/aos/1013203451

[23] Friedman JH. Stochastic gradient boosting. Comput Stat Data Anal. 2002;38(4):367-378. doi:10.1016/S0167-9473(01)00065-2

[24] McCaffrey DF, Griffin BA, Almirall D, Slaughter ME, Ramchand R, Burgette LF. A tutorial on propensity score estimation for multiple treatments using generalized boosted models. Stat Med. 2013;32(19):3388-3414. doi:10.1002/sim.5753 23508673 PMC3710547

[25] Austin PC. Variance estimation when using inverse probability of treatment weighting (IPTW) with survival analysis. Stat Med. 2016;35(30):5642-5655. doi:10.1002/sim.7084 27549016 PMC5157758

[26] Chatelet F, Verillaud B, Chevret S. How to perform prespecified subgroup analyses when using propensity score methods in the case of imbalanced subgroups. BMC Med Res Methodol. 2023;23(1):255. doi:10.1186/s12874-023-02071-8 37907863 PMC10617117

[27] Austin PC, Fine JP. Practical recommendations for reporting Fine-Gray model analyses for competing risk data. Stat Med. 2017;36(27):4391-4400. doi:10.1002/sim.7501 28913837 PMC5698744

[28] Fine JP, Gray RJ. A proportional hazards model for the subdistribution of a competing risk. J Am Stat Assoc. 1999;94(446):496-509. doi:10.1080/01621459.1999.10474144

[29] Bhimani J, O’Connell K, Ergas IJ, . Trends in chemotherapy use for early-stage breast cancer from 2006 to 2019. Breast Cancer Res. 2024;26(1):101. doi:10.1186/s13058-024-01822-9 38872192 PMC11170793

[30] Tarantino P, Leone J, Vallejo CT, . Prognosis and treatment outcomes for patients with stage IA triple-negative breast cancer. NPJ Breast Cancer. 2024;10(1):26. doi:10.1038/s41523-024-00634-6 38575691 PMC10995121

[31] Fisher B, Anderson S, Bryant J, . Twenty-year follow-up of a randomized trial comparing total mastectomy, lumpectomy, and lumpectomy plus irradiation for the treatment of invasive breast cancer. N Engl J Med. 2002;347(16):1233-1241. doi:10.1056/NEJMoa022152 12393820

[32] Ravdin PM, Siminoff IA, Harvey JA. Survey of breast cancer patients concerning their knowledge and expectations of adjuvant therapy. J Clin Oncol. 1998;16(2):515-521. doi:10.1200/JCO.1998.16.2.515 9469335

[33] Duric V, Stockler M. Patients’ preferences for adjuvant chemotherapy in early breast cancer: a review of what makes it worthwhile. Lancet Oncol. 2001;2(11):691-697. doi:10.1016/S1470-2045(01)00559-9 11902540

[34] Du XL, Key CR, Osborne C, Manhken JD, Goodwin JS. Discrepancy between consensus recommendations and actual community use of adjuvant chemotherapy in women with breast cancer. Ann Intern Med. 2003;138(2):90-97. doi:10.7326/0003-4819-138-2-200301210-00009 12529090 PMC2566742

[35] Lyman GH, Dale DC, Crawford J. Incidence and predictors of low dose-intensity in adjuvant breast cancer chemotherapy: a nationwide study of community practices. J Clin Oncol. 2003;21(24):4524-4531. doi:10.1200/JCO.2003.05.002 14673039

[36] Kaplan HG, Malmgren JA, Atwood MK. Triple-negative breast cancer in the elderly: prognosis and treatment. Breast J. 2017;23(6):630-637. doi:10.1111/tbj.12813 28485826

[37] Lichtman SM, Skirvin JA. Pharmacology of antineoplastic agents in older cancer patients. Oncology (Williston Park). 2000;14(12):1743-1755. 11204376

[38] Gajra A, Jeune-Smith Y, Fortier S, . The use and knowledge of validated geriatric assessment instruments among US community oncologists. JCO Oncol Pract. 2022;18(7):e1081-e1090. doi:10.1200/OP.21.00743 35263162

[39] Hurria A, Togawa K, Mohile SG, . Predicting chemotherapy toxicity in older adults with cancer: a prospective multicenter study. J Clin Oncol. 2011;29(25):3457-3465. doi:10.1200/JCO.2011.34.7625 21810685 PMC3624700

[40] Magnuson A, Sedrak MS, Gross CP, . Development and validation of a risk tool for predicting severe toxicity in older adults receiving chemotherapy for early-stage breast cancer. J Clin Oncol. 2021;39(6):608-618. doi:10.1200/JCO.20.02063 33444080 PMC8189621

[41] Cohen HJ, Smith D, Sun CL, ; Cancer and Aging Research Group. Frailty as determined by a comprehensive geriatric assessment-derived deficit-accumulation index in older patients with cancer who receive chemotherapy. Cancer. 2016;122(24):3865-3872. doi:10.1002/cncr.30269 27529755 PMC5138076

[42] Griggs JJ, Hawley ST, Graff JJ, . Factors associated with receipt of breast cancer adjuvant chemotherapy in a diverse population-based sample. J Clin Oncol. 2012;30(25):3058-3064. doi:10.1200/JCO.2012.41.9564 22869890 PMC3732005

[43] Wildiers H, Heeren P, Puts M, . International Society of Geriatric Oncology consensus on geriatric assessment in older patients with cancer. J Clin Oncol. 2014;32(24):2595-2603. doi:10.1200/JCO.2013.54.8347 25071125 PMC4876338

[44] Mohile SG, Dale W, Somerfield MR, . Practical assessment and management of vulnerabilities in older patients receiving chemotherapy: ASCO guideline for geriatric oncology. J Clin Oncol. 2018;36(22):2326-2347. doi:10.1200/JCO.2018.78.8687 29782209 PMC6063790

[45] Schmid P, Cortes J, Dent R, ; KEYNOTE-522 Investigators. Event-free survival with pembrolizumab in early triple-negative breast cancer. N Engl J Med. 2022;386(6):556-567. doi:10.1056/NEJMoa2112651 35139274

[46] Schmid P, Cortes J, Dent R, ; KEYNOTE-522 Investigators. Overall survival with pembrolizumab in early-stage triple-negative breast cancer. N Engl J Med. 2024;391(21):1981-1991. doi:10.1056/NEJMoa2409932 39282906

[47] Schmid P, Cortes J, Pusztai L, ; KEYNOTE-522 Investigators. Pembrolizumab for early triple-negative breast cancer. N Engl J Med. 2020;382(9):810-821. doi:10.1056/NEJMoa1910549 32101663

