# Supplementary material for: Adjuvant Chemotherapy Outcomes in Older Adults With Nonmetastatic Triple-Negative Breast Cancer
Source: JAMA Netw Open. 2026 Apr 6;9(4):e265061. doi: 10.1001/jamanetworkopen.2026.5061 (PMC13054624; doi:10.1001/jamanetworkopen.2026.5061)
Supplement: Supplement 1. — eTable 1. Balance Diagnostics Before and After Inverse Probability of Treatment Weighting (IPTW) eTable 2. Use of Adjuvant Chemotherapy in Older Patients With TNBC From 2010 to 2021 eTable 3. Univariate and Multivariable Analysis for Breast Cancer-Specific Survival in Older Patients With Stages I-III TNBC After Inverse Probability of Treatment Weighting (IPTW) Analysis eTable 4. Univariate and Multivariable Analysis for Overall Survival in Older Patients With Stages I-III TNBC After Inverse Probability of Treatment Weighting (IPTW) Analysis eTable 5. Benefit of Chemotherapy in Overall Survival for Older Patients With Stages I-III TNBC After Inverse Probability of Treatment Weighting (IPTW) Analysis eFigure 1. Balance Diagnostic Before and After IPTW eFigure 2. Strobe Diagram eFigure 3. Cumulative Incidence of Non–Breast Cancer Death by Chemotherapy Use in Older Patients With Stages I-III TNBC eFigure 4. Overall Survival in Older Patients With Stages I-III TNBC [file jamanetwopen-e265061-s001.pdf]

Supplementary Online Content

Anampa JD, Avila J, Brodsky M, et al. Adjuvant chemotherapy outcomes in older adults with nonmetastatic triple-negative breast cancer. *JAMA Netw Open.* 2026;9(4):e265061. doi:10.1001/jamanetworkopen.2026.5061

eTable 1. Balance Diagnostics Before and After Inverse Probability of Treatment Weighting (IPTW)

eTable 2. Use of Adjuvant Chemotherapy in Older Patients With TNBC From 2010 to 2021

eTable 3. Univariate and Multivariable Analysis for Breast Cancer-Specific Survival in Older Patients With Stages I-III TNBC After Inverse Probability of Treatment Weighting (IPTW) Analysis

eTable 4. Univariate and Multivariable Analysis for Overall Survival in Older Patients With Stages I-III TNBC After Inverse Probability of Treatment Weighting (IPTW) Analysis

eTable 5. Benefit of Chemotherapy in Overall Survival for Older Patients With Stages I-III TNBC After Inverse Probability of Treatment Weighting (IPTW) Analysis

eFigure 1. Balance Diagnostic Before and After IPTW

eFigure 2. Strobe Diagram

eFigure 3. Cumulative Incidence of Non–Breast Cancer Death by Chemotherapy Use in Older Patients With Stages I-III TNBC

eFigure 4. Overall Survival in Older Patients With Stages I-III TNBC

This supplementary material has been provided by the authors to give readers additional information about their work.

© 2026 Anampa JD et al. *JAMA Network Open*.

eTable 1. Balance Diagnostics Before and After Inverse Probability of Treatment Weighting (IPTW)

Variable	Unweighted			Weighted		
	Mean PS chemotherapy	Mean PS No chemo	Standardized effect size	Mean PS chemotherapy	Mean PS no chemo	Standardized effect size
Age Group	1.12	1.53	-0.76	1.32	1.36	-0.07
Race	1.55	1.56	-0.01	1.55	1.55	0.00
Stage	1.68	1.66	0.02	1.66	1.66	-0.00
Tumor Grade	1.82	1.76	0.15	1.79	1.78	0.02
Radiation	1.60	1.43	0.34	1.51	1.50	0.01
Surgery	1.33	1.45	-0.24	1.39	1.39	-0.00
Marital status	2.47	2.38	0.13	2.42	2.41	0.00
Rurality	1.13	1.13	0.01	1.13	1.12	0.01
Year of diagnosis	1.86	1.75	0.13	1.79	1.80	-0.00
Income	1.64	1.64	0.01	1.64	1.64	-0.00

Abbreviations: Chemo, chemotherapy; PS, propensity score.

eTable 2. Use of Adjuvant Chemotherapy in Older Patients With TNBC From 2010 to 2021

year	All patients (>70 years)		70 - 79 years		80 - 89 years		≥90 years	
	**Chemo**	**No chemo**	**Chemo**	**No chemo**	**Chemo**	**No chemo**	**Chemo**	**No chemo**
2010	179 (36%)	321 (64%)	151 (44%)	189 (56%)	27 (18%)	119 (82%)	1 (7%)	13 (93%)
2011	185 (37%)	315 (63%)	163 (50%)	165 (50%)	22 (14%)	140 (86%)	0 (0%)	10 (100%)
2012	217 (41%)	307 (59%)	184 (53%)	161 (47%)	32 (21%)	124 (79%)	1 (4%)	22 (96%)
2013	220 (46%)	263 (54%)	197 (59%)	135 (41%)	23 (17%)	114 (83%)	0 (0%)	14 (100%)
2014	198 (41%)	287 (59%)	181 (56%)	143 (44%)	16 (11%)	128 (89%)	1 (6%)	16 (94%)
2015	219 (47%)	249 (53%)	195 (59%)	138 (41%)	24 (19%)	100 (81%)	0 (0%)	11 (100%)
2016	206 (43%)	273 (57%)	187 (58%)	133 (42%)	19 (13%)	123 (87%)	0 (0%)	17 (100%)
2017	213 (44%)	270 (56%)	187 (55%)	156 (45%)	24 (20%)	96 (80%)	2 (10%)	18 (90%)
2018	220 (50%)	224 (50%)	194 (63%)	113 (37%)	26 (21%)	99 (79%)	0 (0%)	12 (100%)
2019	245 (48%)	263 (52%)	213 (63%)	125 (37%)	32 (21%)	117 (79%)	0 (0%)	21 (100%)
2020	189 (46%)	219 (54%)	168 (63%)	97 (37%)	21 (16%)	113 (84%)	0 (0%)	9 (100%)
2021	218 (49%)	230 (51%)	189 (61%)	119 (39%)	29 (23%)	98 (77%)	0 (0%)	13 (100%)

Abbreviations: TNBC, Triple-Negative Breast Cancer; SEER, Surveillance, Epidemiology, and End Results; Chemo, chemotherapy.

eTable 3. Univariate and Multivariable Analysis for Breast Cancer-Specific Survival in Older Patients With Stages I-III TNBC After Inverse Probability of Treatment Weighting (IPTW) Analysis

© 2026 Anampa JD et al. *JAMA Network Open*.

Characteristics	Univariate			Multivariable		
	**HR**	**95% CI**	**p-value**	**HR**	**95% CI**	**p-value**
**Age (years)**						
70 – 79	Reference			Reference		
80 – 89	1.79	1.50 – 2.13	<0.01	1.31	1.09 – 1.58	0.01
≥90	4.03	2.95 – 5.52	<0.01	1.99	1.40 – 2.84	<0.01
**Race/ethnicity**						
NH White	Reference			Reference		
NH Black	1.03	0.84 – 1.27	0.77	0.96	0.77 – 1.21	0.76
Hispanics	1.02	0.76 – 1.38	0.88	0.95	0.69 – 1.30	0.74
NH others	0.73	0.51 – 1.04	0.08	0.69	0.48 – 0.99	0.04
**Year of diagnosis**						
2010-2015	Reference			Reference		
2016-2017	0.83	0.66 – 1.04	0.10	0.97	0.78 – 1.23	0.85
2018-2021	0.76	0.59 – 0.97	0.03	0.69	0.54 – 0.88	0.01
**Marital status**						
Never married	Reference			Reference		
Prior married	1.15	0.88 – 1.51	0.28	1.17	0.89 – 1.55	0.26
Married	0.97	0.74 – 1.28	0.84	1.22	0.91 – 1.63	0.19
Unknown	0.59	0.36 – 0.97	0.04	0.73	0.45 – 1.20	0.22
**Rurality**						
Metropolitan	Reference			Reference		
Non-metro	1.12	0.90 – 1.39	0.33	1.08	0.83 – 1.41	0.58
**Income**						
< $70k	Reference			Reference		
≥ $70k	0.83	0.71 – 0.97	0.02	0.91	0.74 – 1.11	0.35
**Tumor Grade**						
Well/moderately differentiated	Reference			Reference		
Poorly/undifferentiated	1.89	1.52 – 2.37	<0.01	1.41	1.13 – 1.76	0.01
**Surgery**						
BCS	Reference			Reference		
Mastectomy	2.45	2.09 – 2.87	<0.01	1.18	0.97 – 1.43	0.10
**Chemotherapy**						
No	Reference			Reference		
Yes	0.74	0.63 – 0.87	<0.01	0.69	0.58 – 0.82	<0.01
**Radiation**						
No	Reference			Reference		
Yes	0.56	0.47 – 0.65	<0.01	0.70	0.58 – 0.85	<0.01
**Stage**						
Stage I	Reference			Reference		
Stage II	3.07	2.51 – 3.76	<0.01	2.63	2.14 – 3.23	<0.01
Stage III	10.24	8.25 – 12.72	<0.01	8.60	6.74 – 10.96	<0.01

Abbreviations: TNBC, Triple-negative Breast Cancer; HR, Hazard Ratios; CI, Confidence Interval; NH, Non-Hispanic; k, Thousand Dollars; BCS, Breast-conserving Surgery.

eTable 4. Univariate and Multivariable Analysis for Overall Survival in Older Patients With Stages I-III TNBC After Inverse Probability of Treatment Weighting (IPTW) Analysis

© 2026 Anampa JD et al. *JAMA Network Open*.

Characteristics	Univariate HR	95% CI	p-value	Multivariable HR	95% CI	p-value
**Age (years)**						
70 – 79	Reference			Reference		
80 – 89	1.97	1.76 – 2.21	<0.01	1.57	1.40 – 1.77	<0.01
≥90	4.64	3.79 – 5.69	<0.01	2.38	1.92 – 2.94	<0.01
**Race/ethnicity**						
NH White	Reference			Reference		
NH Black	1.14	0.99 – 1.31	0.05	1.04	0.90 – 1.20	0.58
Hispanics	1.11	0.92 – 1.33	0.28	1.03	0.85 – 1.24	0.79
NH others	0.79	0.63 – 0.98	0.03	0.73	0.58 – 0.91	0.01
**Year of diagnosis**						
2010-2015	Reference			Reference		
2016-2017	0.95	0.82 – 1.11	0.53	1.07	0.92 – 1.25	0.35
2018-2021	0.79	0.66 – 0.95	0.01	0.76	0.63 – 0.91	0.01
**Marital status**						
Never married	Reference			Reference		
Prior married	1.16	0.97 – 1.37	0.10	1.13	0.94 – 1.36	0.20
Married	0.77	0.65 – 0.92	0.01	0.92	0.76 – 1.13	0.44
Unknown	0.86	0.65 – 1.14	0.30	0.98	0.73 – 1.30	0.88
**Rurality**						
Metropolitan	Reference			Reference		
Non-metro	1.06	0.91 – 1.23	0.46	1.01	0.86 – 1.20	0.87
**Income**						
< $70k	Reference			Reference		
≥ $70k	0.83	0.75 – 0.92	<0.01	0.87	0.77 – 0.98	0.03
**Tumor Grade**						
Well/moderately differentiated	Reference			Reference		
Poorly/undifferentiated	1.63	1.43 – 1.87	<0.01	1.37	1.19 – 1.57	<0.01
**Surgery**						
BCS	Reference			Reference		
Mastectomy	1.94	1.75 – 2.14	<0.01	1.13	0.99 – 1.30	0.07
**Chemotherapy**						
No	Reference			Reference		
Yes	0.58	0.52 – 0.65	<0.01	0.55	0.49 – 0.62	<0.01
**Radiation**						
No	Reference			Reference		
Yes	0.61	0.55 – 0.67	<0.01	0.76	0.66 – 0.87	<0.01
**Stage**						
Stage I	Reference			Reference		
Stage II	2.14	1.91 – 2.40	<0.01	1.86	1.65 – 2.10	<0.01
Stage III	5.30	4.53 – 6.19	<0.01	4.43	3.73 – 5.25	<0.01

Abbreviations: TNBC, Triple-negative Breast Cancer; HR, Hazard Ratios; CI, Confidence Interval; NH, Non-Hispanic; k, Thousand Dollars; BCS, Breast-conserving Surgery.

eTable 5. Benefit of Chemotherapy in Overall Survival for Older Patients With Stages I-III TNBC After Inverse Probability of Treatment Weighting (IPTW) Analysis

© 2026 Anampa JD et al. *JAMA Network Open*.

Subgroup	No. of Patients	No. of Events	HR (95% CI)	5-Year OS no chemo	5-Year OS chemo	p for interaction
**Overall**	5730	1980	0.55 (0.49-0.62)	61%	76%	N/A
**Age**						0.65
70-79	3883	1044	0.55 (0.48-0.63)	68%	81%	
80-89	1666	809	0.53 (0.43-0.66)	49%	65%	
>=90	181	127	0.92 (0.28-2.80)	29%	62%	
**Race**						0.53
NH White	3930	1376	0.51 (0.45-0.58)	60%	77%	
NH Black	875	314	0.58 (0.44-0.76)	60%	73%	
Hispanic	481	172	0.70 (0.46-1.07)	63%	68%	
Other	444	118	0.52 (0.31-0.89)	70%	84%	
**Tumor Grade**						0.62
Well/moderately differentiated	1244	343	0.59 (0.43-0.81)	74%	83%	
Poorly/undifferentiated	4486	1637	0.55 (0.48-0.62)	57%	75%	
**Stage**						0.60
Stage I	2698	607	0.59 (0.48-0.72)	77%	88%	
Stage II	2227	913	0.56 (0.48-0.66)	54%	73%	
Stage III	805	460	0.51 (0.40-0.66)	22%	45%	
**Radiation**						0.45
No	2821	1218	0.58 (0.50-0.67)	55%	70%	
Yes	2909	762	0.52 (0.43-0.62)	67%	82%	
**Surgery**						0.72
Mastectomy	2261	1068	0.56 (0.47-0.66)	50%	66%	
BCS	3469	912	0.53 (0.45-0.62)	68%	83%	

Models were adjusted for race, age, cancer stage, tumor grade, radiation, type of surgery, household income, marital status, rurality, and year of diagnosis. Abbreviations: CI, confidence interval; HR, hazard ratio; OS, Overall Survival; NH, non-Hispanic.

© 2026 Anampa JD et al. *JAMA Network Open*.

eFigure 1. Balance Diagnostic Before and After IPTW

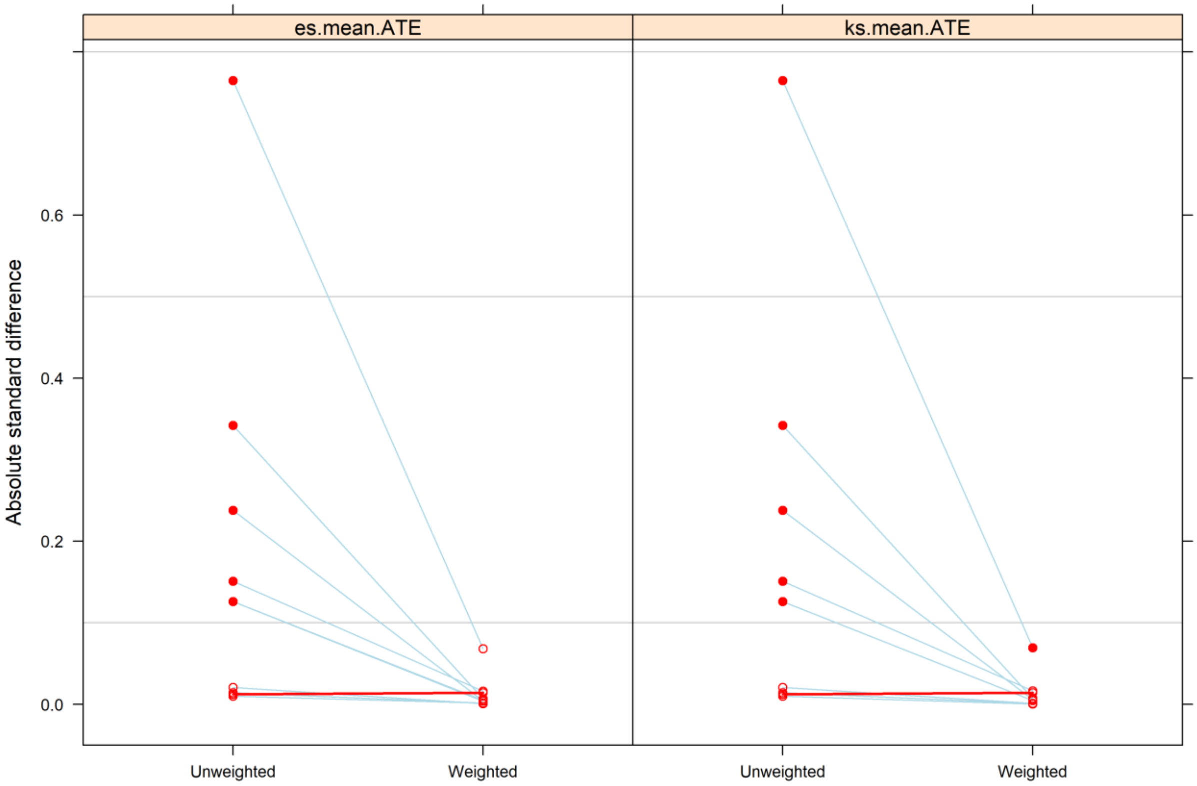

Abbreviations: ATE, (Average Treatment Effect; ks, Kolmogorov-Smirnov statistic; es, standardized effect size; IPWT, Inverse probability of treatment weighting.

© 2026 Anampa JD et al. *JAMA Network Open*.

eFigure 2. Strobe Diagram

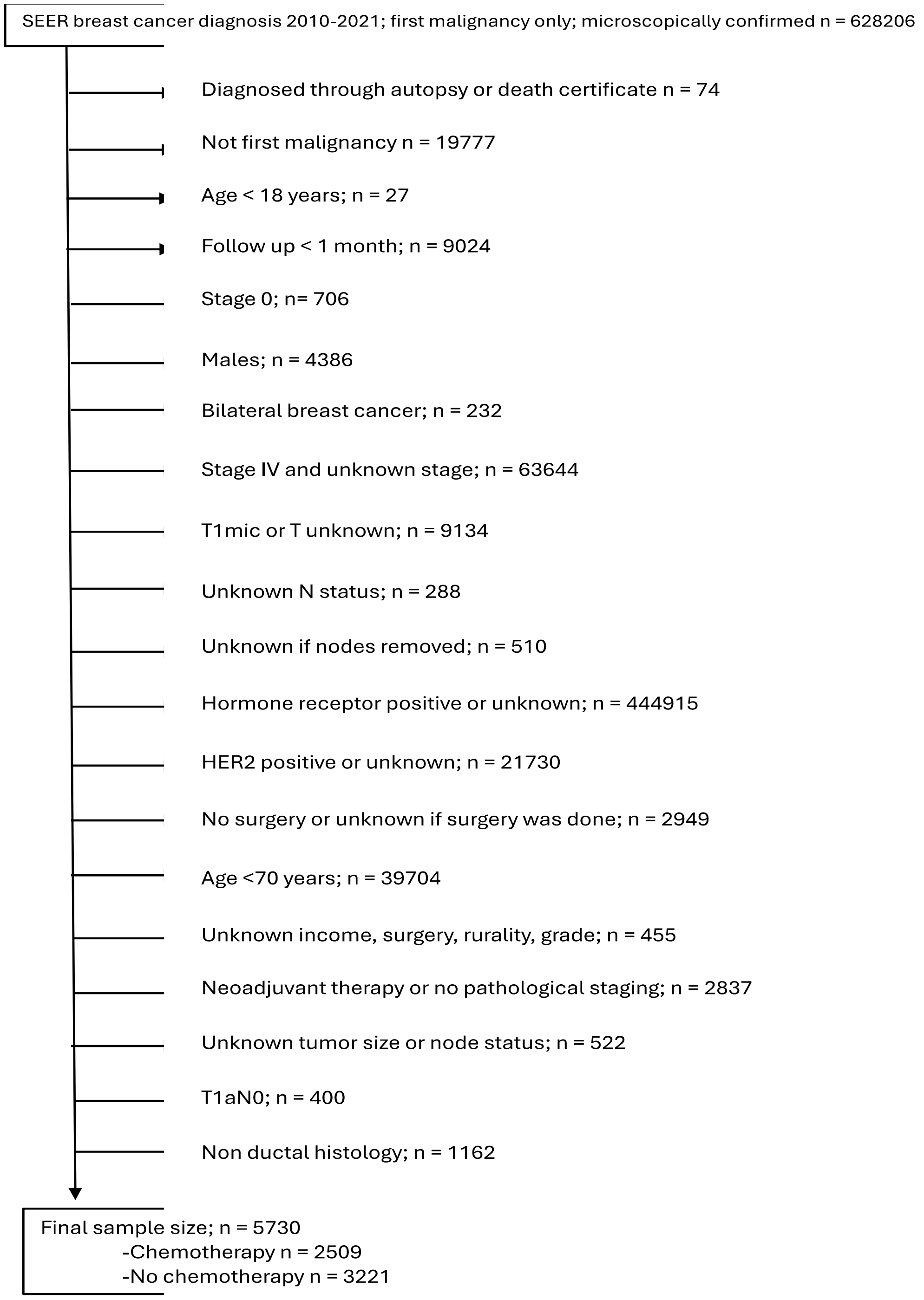

Abbreviations: HER2, Human epidermal growth factor receptor 2; n, number; N, nodal stage; n, number; T, tumor size stage.

© 2026 Anampa JD et al. *JAMA Network Open*.

eFigure 3. Cumulative Incidence of Non–Breast Cancer Death by Chemotherapy Use in Older Patients With Stages I-III TNBC

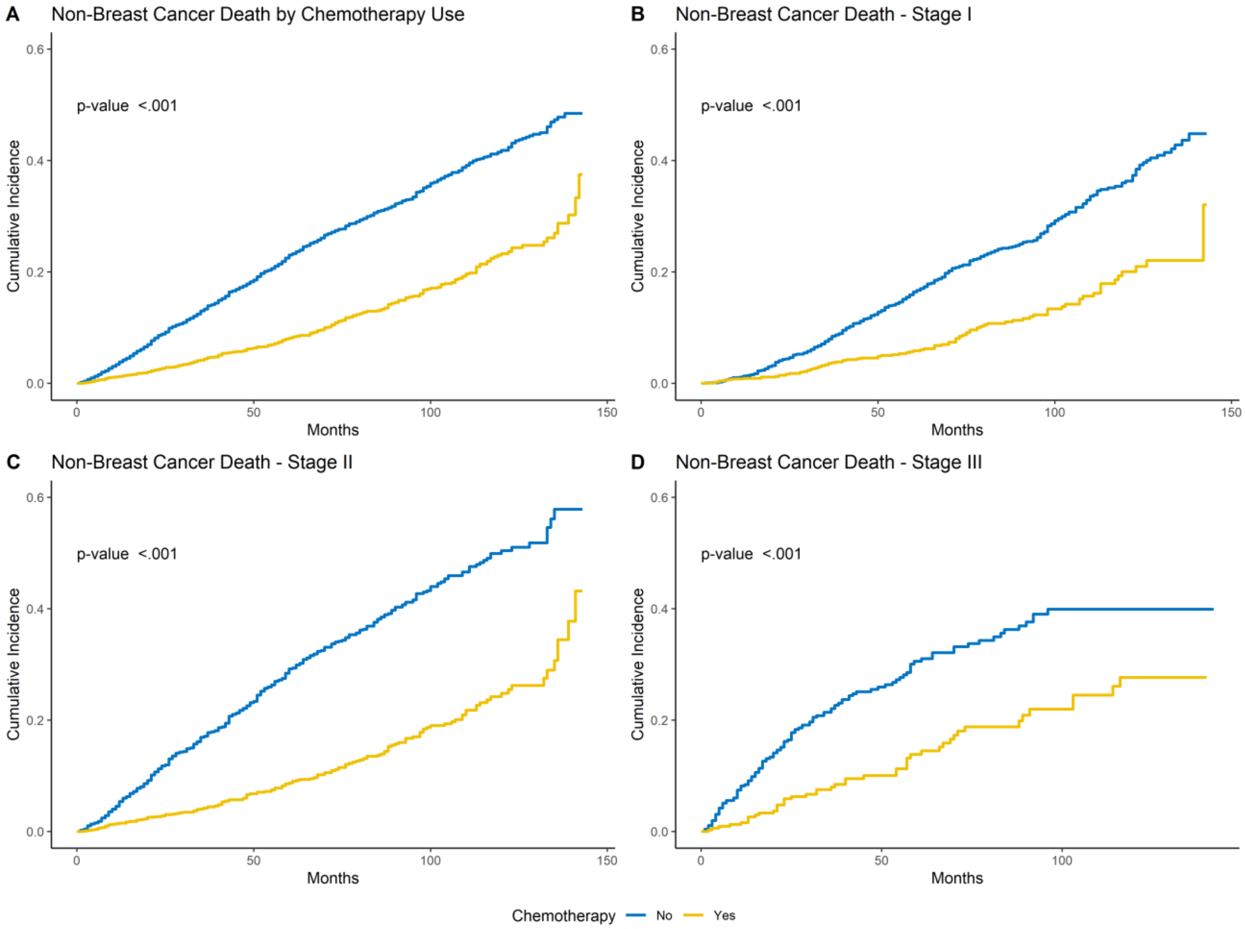

A) All patients, B) Stage I TNBC, C) Stage II TNBC, D) Stage III TNBC. Abbreviations: TNBC, Triple-negative Breast Cancer. Yellow line: chemotherapy group, Blue line: no chemotherapy group.

© 2026 Anampa JD et al. *JAMA Network Open*.

eFigure 4. Overall Survival in Older Patients With Stages I-III TNBC

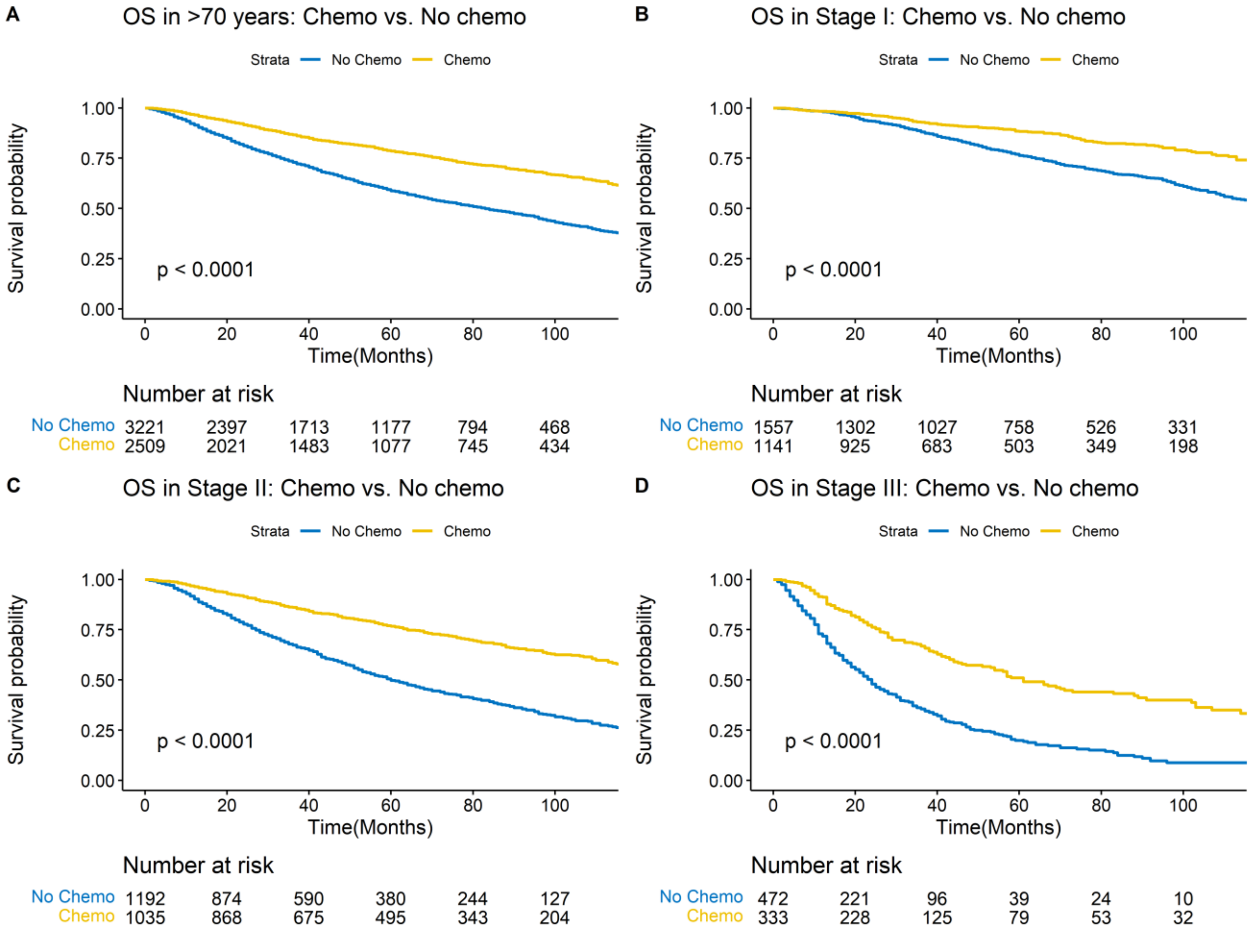

A) All patients, B) Stage I TNBC, C) Stage II TNBC, C) Stage III TNBC. Abbreviations: OS, Overall Survival; TNBC, Triple Negative Breast Cancer; Chemo, chemotherapy. Yellow line: chemotherapy group, Blue line: no chemotherapy group.

© 2026 Anampa JD et al. *JAMA Network Open*.
