# Supplementary material for: Adjuvant Chemotherapy Outcomes in Older Adults With Nonmetastatic Triple-Negative Breast Cancer
Source: JAMA Netw Open. 2026 Apr 6;9(4):e265061. doi: 10.1001/jamanetworkopen.2026.5061 (PMC13054624; doi:10.1001/jamanetworkopen.2026.5061)
Supplement: Supplement 2. — Data Sharing Statement [file jamanetwopen-e265061-s002.pdf]

Data Sharing Statement

Anampa. Adjuvant Chemotherapy Outcomes in Older Adults With Nonmetastatic Triple-Negative Breast Cancer. *JAMA Netw Open*. Published April 06, 2026.
doi:10.1001/jamanetworkopen.2026.5061

Data
Data available: Yes
Data types: Deidentified participant data
How to access data: Deidentified data is available from the SEER database.
When available: With publication

Supporting Documents
Document types: Other (please specify)
Additional Information: de-identified data
How to access documents: deidentified data from SEER
When available: With publication

Additional Information
Who can access the data: anyone requesting data.
Types of analyses: for research purposes.
Mechanisms of data availability: after proposal approval by SEER
